# Seasonal Variations and Factors that Influence Diverticular Bleeding in the United States of America

**DOI:** 10.34172/jrhs.2023.112

**Published:** 2023-06-29

**Authors:** Lindsay Talemal, Kausthubha Yaratha, Brian V. Monahan, Daohai Yu, Xiaoning Lu, Juan Lucas Poggio

**Affiliations:** ^1^Temple University, Lewis Katz School of Medicine, Philadelphia, PA, USA; ^2^Temple University Hospital, Department of Surgery, Philadelphia, PA, USA; ^3^Center for Biostatistics and Epidemiology, Department of Biomedical Education and Data Science, Lewis Katz School of Medicine at Temple University, Philadelphia, PA, USA

**Keywords:** Diverticular diseases, Diverticulitis, Seasons, Gastrointestinal hemorrhage

## Abstract

**Background:** Seasonal variation in hospitalizations for diverticulitis has a sinusoidal pattern, peaking in summer. Little is known about seasonal, regional trends, and risk factors associated with hospital admissions regarding diverticular bleeding in the United States.

**Study Design:** Cross-sectional population database review using the healthcare cost and utilization project’s national inpatient sample.

**Methods:** Patients that had diagnoses of diverticulitis with bleeding or diverticulosis with bleeding admitted from January 1, 2015, through December 31, 2017, were identified and stratified by month and season. Then, the potential effects of region, age, gender, race, and patient risk factors on seasonal admissions for diverticular bleeding were explored, and data were analyzed in SAS and presented in Excel using chi-square and Kruskal-Wallis for categorical and continuous variables, respectively.

**Results:** Of the 54191 hospitalized cases for diverticular bleeding, the peak and the lowest seasons were spring and summer (25.5% vs. 24.2%, *P*<0.0001). A significant seasonal pattern in comorbidities was also identified, and those with diabetes (*P*<0.0001), hypertension (HTN) (*P*<0.0001), obesity (*P*<0.0001), and those on anticoagulants (*P*=0.016) all had more bleeding events in the spring. This was noted across US regions, gender, race, and age. Eventually, the southern region had the most admissions for diverticular bleeding at 40.9% (*P*<0.0001).

**Conclusion:** A better understanding of these seasonal and regional trends may provide a mechanism to identify a potential trigger for diverticular bleeding events. This helps identify individuals at greatest risk for hospitalization, as well as prepare hospitals to allocate supplies appropriately during the seasons.

## Background

 Diverticular disease, a common condition affecting both men and women, is prevalent in 10% and 50% of people over age 40 and 60, respectively. Diverticulitis has been described to fluctuate throughout the calendar year.^1^ Diverticular disease exists on a disease spectrum and includes complications such as perforation, abscess, and bleeding.

 One such complication, namely, diverticular bleeding, is the source of 17%-40% of lower gastrointestinal (GI) hemorrhage in adults, which makes it the single most common cause of lower GI bleeding.^2-4^ In a study of 1593 patients with diverticulosis, severe life-threatening diverticular hemorrhage occurred in 3.1% of patients.^5^

 Over the past several decades, diverticular disease has increased in prevalence for unclear reasons. There exist many hypotheses about this increase, including diets heavier in processed foods lacking fiber, vitamin D deficiency, obesity, and non-steroidal, anti-inflammatory drug use.^1,6-8^ With no specific identified cause for diverticular disease, let alone diverticular bleeding, this investigation decided to look for seasonal patterns and potential epidemiologic factors associated with the acute episodes of diverticular bleeding that lead to hospitalization in the United States (US). Seasonal patterns have been identified in several acute inflammatory conditions, particularly other acute inflammatory conditions of the GI tract such as appendicitis, and thus the authors expected that diverticular bleeding may follow some seasonal patterns and have epidemiological association.^9^ This, in turn, could aid in further establishing etiologic triggers for acute diverticular bleeding.

## Methods

###  Data collection

 Patient data were obtained using the National Inpatient Sample (NIS), the largest source of all-payer hospital discharge information in the US which includes admission-level data from all the participating hospitals in 47 US states.^10^ The NIS provides information regarding admission month, geographical region, discharge diagnoses, procedure codes, gender, race, and comorbidities.

 The inclusion criteria were admissions between January 1, 2015, through December 31, 2017, via the Healthcare Cost and Utilization Project of the Agency for Healthcare Research and Quality. Hospital region was obtained from the NIS and defined as Northeast (New England and Middle Atlantic), Southeast (South Atlantic, East South Central, and West South Central), Midwest (West North Central, East North Central), and West (Pacific and Mountain). Since this study utilized and analyzed publicly available, deidentified data, it was deemed exempt from the Institutional Review Board.

###  Study population

 A cross-sectional sample of all US inpatient admissions and discharges from January 1, 2015, to December 31, 2017, was employed in this study. Using both the standard International Classification of Disease, Ninth Edition, Clinical Modification (ICD-9-CM) codes and the International Classification of Disease, Tenth Edition, Clinical Modification (ICD-10-CM) codes, all adult hospitalization cases (age ≥ 18) with any diagnosis code corresponding to diverticulosis with bleeding or diverticulitis with bleeding were included in the analysis.

 The seasonality was determined by looking at the month of each hospitalization admission date. Using the meteorological definition of seasons in the northern hemisphere, Spring, Summer, Fall, and Winter were defined by the month March 1 through May 31, June 1 through August 31, September 1 through November 30, and December 1 through February 28, respectively. The metrics of obesity, hypertension (HTN), anticoagulant use, and diabetes were defined by the NIS. HTN was defined as systolic blood pressure > 140 or diastolic blood pressure > 90.^10^ Obesity was defined as body mass index (BMI) > 30.^10^

###  Study outcomes and statistical analysis

 The primary outcome analyzed by this study was the hospitalization rate of diverticular bleeding assessed throughout the seasons using the Kruskal-Wallis test and chi-square for continuous and categorical variables, respectively. Patient characteristics such as age, race, gender, and the US region were assessed as well. The rates of admission comorbidities, including diabetes, coagulation disorders, HTN, obesity, and patients on anticoagulants, were all determined both seasonally and regionally and underwent comparison.

**Figure 1:**
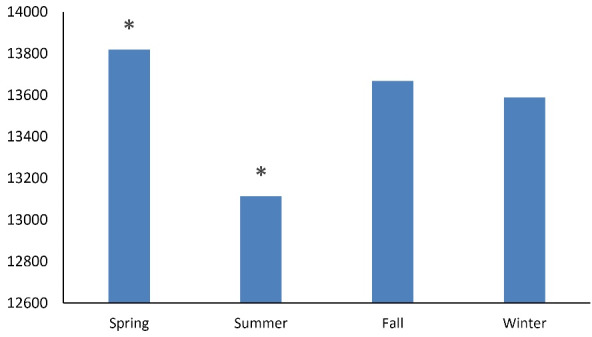
Total seasonal overall admissions for diverticular bleeding in USA 1/1/15-12/31/17. *Note*. ^*^P < 0.05

**Figure 2:**
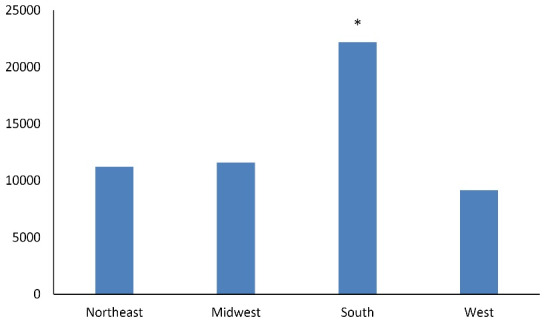
Total regional overall admissions for diverticular bleeding USA 1/1/15-12/31/17. *Note*. ^*^P < 0.05

**Figure 3:**
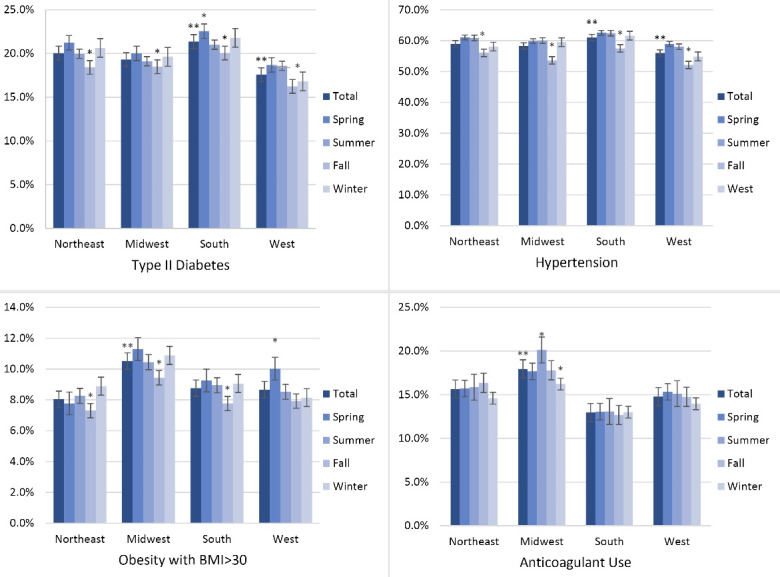
Percent regional overall admissions by season for diverticular bleeding in USA 1/1/15-12/31/17 by Co-morbid Conditions. *Note*. BMI: Body mass index. ^*^P < 0.05 for regional groups, ^**^P < 0.05 for total groups

**Figure 4:**
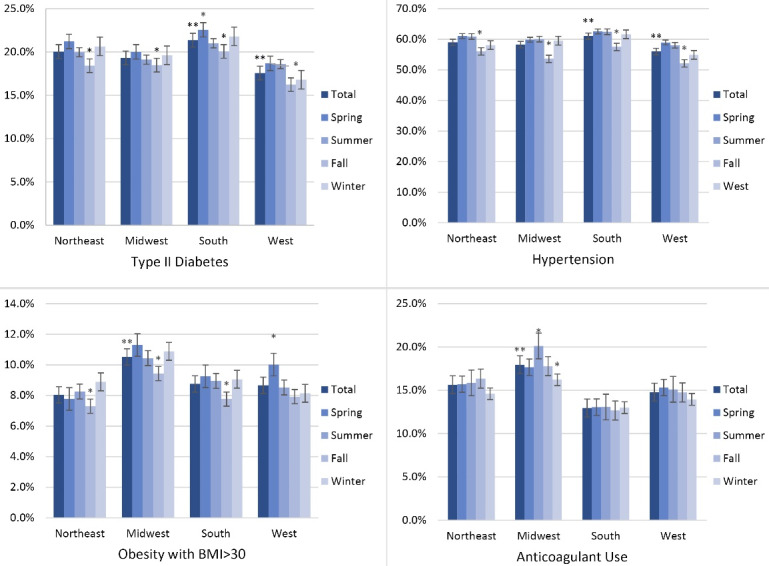
Percent seasonal overall admissions by region for diverticular bleeding in USA 1/1/15-12/31/17 by Co-morbid Conditions. *Note*. BMI: Body mass index. ^*^P < 0.05 for seasonal groups, ^**^P < 0.05 for total groups

 Admission incidence by season was compared with patient characteristics using Rao-Scott chi-square tests. Group analyses were also performed to adjust for gender, age, race, HTN, diabetes, obesity, aspirin use, and anticoagulant use. All the statistical analyses were conducted using SAS, and figures were created using Microsoft Excel. Data were tabulated and presented by regional and seasonal analyses (Figures 1 and 2) and then by comorbid conditions studied (Figures 3 and 4). Intergroup statistical significance was performed by region and season. Additional total group statistical significance was performed as well. For example, in Figure 3 in the southern cohort, the spring had a statistically significant increased prevalence of patients with diabetes presenting with diverticular bleeding (indicated by *). Additionally, the southern region had a statistically significant increased prevalence of patients with diabetes presenting with diverticular bleeding across all regions.

## Results

###  Patient characteristics

 The population consisted of 65.4% White, 20.1% Black or African American, and 6.7% Hispanic or Latino, as well as 52.1% women. The age range of admission was 18-90 years, and the average age of admission was 77 years old. However, out of the entire patient population, 72.3% were above the age of 69.

 When all admissions were aggregated, spring demonstrated the greatest number of diverticular bleeding admissions (13 823, 25.5%), followed by fall (13 666, 25.2%), winter (13 590, 25.1%), and summer (13 112, 24.2%) (*P* < 0.0001, Figures 1-2). By region, the South accounted for the most admissions (40.9%), followed by the Midwest (21.4%), the Northeast (20.7%), and the West (16.9%); this was significant (*P* < 0.05, Figure 2).

 Regional and seasonal patterns were identified. Patients in the southern region who presented with diverticular bleeding were more likely to have comorbid diabetes and HTN, while those in the Midwestern region were more likely to have BMI > 30 or use anticoagulants (*P* < 0.05, Figure 3). Patients with diverticular bleeding who had comorbid diabetes, coagulation disorders, HTN, and obesity were presented more often in the spring (*P* < 0.05). Patients using anticoagulants were less prevalent in the winter (Figure 4).

 Next, the risk difference of patients with co-morbidities was calculated compared to the total number of patients for the season studied to identify if differences in patients with co-morbid conditions were just due to the observed seasonal variations (Table 1). Based on the results, patients with obesity were 6.2% more likely to present in the spring with diverticular bleeding compared to the average number of patients with obesity who presented with diverticular bleeding throughout the year.

**Table 1:** Risk difference for presenting to hospital with diverticular bleeding by season for the examined co-morbidities

	Spring (%)	Summer (%)	Fall (%)	Winter (%)
Diabetes	5.3	-0.2	-6.4	1.2
Hypertension	3.2	2.8	-6.3	0.3
Obesity	6.2	1.0	-10.3	3.1
Anticoagulant use	0.6	4.4	-0.1	-4.9

*Note.* A positive number indicates an increased risk, while a negative number indicates a decreased risk.

## Discussion

 Data analysis suggested a modest but statistically significant seasonal variation in the incidence of hospital admissions with a peak in the spring. Based on the finding, patients presented with diverticular bleeding from the southern regions were more likely to have co-morbid diabetes or HTN, whereas cases presented from the Midwestern regions were more likely to have a BMI > 30 or consume anticoagulants (Figure 3). The data (Figure 4) also demonstrated that patients with certain risk factors are more likely to present in the spring months (diabetes, HTN, and obesity) and less likely to present in the winter (anticoagulant use).

 Although the finding related to seasonal variations in diverticular bleeding is novel, prior observations revealed that diverticulitis, a related but distinct pathology is more prevalent in the summer.^11-13^ It is unclear why diverticulitis and diverticular bleeding have distinct seasonality prevalence. Diverticulitis usually peaks in the summer, while our data indicated a slight prevalence in the winter months. Multiple studies reported that the inflammatory pathogenesis of diverticulitis while diverticular bleeding is associated with the distorted microvascular architecture of the diverticular vasa recta. Diseases altering the microvascular architecture, including diabetes, HTN, and now possibly obesity, have been linked to diverticular bleeding.^12-14^

 Another possible explanation for the seasonality of bleeding events found in the literature attributes the events to seasonal changes in diet, physical activity, or medication use, which may trigger the onset of a bleeding event.^6,15-18^ Some studies have shown that diets poor in fresh fruits and vegetables lead to vitamin deficiencies which could predispose to bleeding.^6,17,18^ Similarly, seasonal changes in UV radiation were thought to influence diverticulosis, diverticulitis, and possible bleeding, potentially due to vitamin D.^8^

 Additionally, the obtained data demonstrated that patients with BMI > 30 have an increased prevalence of diverticular bleeding events in the spring. Obesity has been known to create a pro-inflammatory state and cause disruptions in the normal regulation of the gut microbiome.^18^ Some studies have also reported a link between diverticular disease and obesity.^14,19,20^

 Overall, the data suggest that there is a greater risk of hospitalization from diverticular bleeding in the springtime. This information may help identify patients who are at high risk and allow patients with known diverticulosis or diverticulitis to be counseled about what signs and symptoms to look out for in terms of a bleeding event and take measures to lessen the impact of diverticular bleeding during the spring when they are at greatest risk.

 This study has several limitations. The NIS is an administrative database and is therefore subject to coding errors. The NIS also is not as good at picking up un-billed co-morbid conditions such as obesity. The regional data of broad strokes do not account for different variations in climate such as the difference between Colorado and California, which are both in the “West” category. The data are also admission-specific which could lead to oversampling if by chance a patient had a readmission or was transferred to another hospital. It is also impossible to analyze all the potential biases and confounding factors in a retrospective observational study such as the current one. Additionally, while the observed data were statistically significant, the absolute differences were small totaling only a difference of around 700 patients between the highest and lowest seasons. These data are likely the best ones used for identifying modifiable risk factors for diverticular bleeding such as HTN, diabetes, and obesity. Finally, this observational retrospective study cannot prove causation for the observed trends.

**Highlights**

- Diverticular bleeding admissions peaked in the spring season in the United States (the US).
- Diverticular bleeding admissions were more likely to occur in the southern region of the US.
- Patients with co-morbid conditions such as diabetes, hypertension, and obesity were also more likely to present in the spring season in the US.

## Conclusion

 The data suggest significant seasonal variations in hospital admissions for diverticular bleeding, with a peak in the spring. Seasonal and regional trends in hospitalizations due to diverticular bleeding may help identify modifiable risk factors which can improve diagnostic and treatment outcomes for patients by allowing for more targeted identification of vulnerable patients. Patients with risk factors such as HTN, diabetes, obesity, and anticoagulant use are also at risk in the spring season. Further work is necessary to identify granular changes at the local climate level and better characterize co-morbidity risk not captured in the NIS.

## Acknowledgements

 The authors would like to thank the Temple University Hospital Department of Surgery for the research support and the Temple University Lewis Katz School of Medicine for their research support.

## Authors’ Contribution

**Conceptualization: **Juan Poggio.

**Data curation: **Lindsay Talemal, Kausthubha Yaratha.

**Formal analysis: **Daohai Yu, Xiaoning Lu.

**Funding acquisition: **Juan Poggio.

**Investigation: **Brian Monahan, Juan Poggio.

**Methodology: **Daohai Yu, Xiaoning Lu, Juan Poggio.

**Project administration:** Brian Monahan, Juan Poggio.

**Resources: **Lindsay Talemal, Kausthubha Yaratha, Brian Monahan, Daohai Yu, Xiaoning Lu.

**Software: **Daohai Yu, Xiaoning Lu.

**Supervision: **Juan Poggio.

**Validation: **Daohai Yu, Xiaoning Lu, Brian Monahan, Juan Poggio.

**Visualization: **Lindsay Talemal, Brian Monahan.

**Writing–original draft: **Lindsay Talemal, Kausthubha Yaratha, Brian Monahan.

**Writing–review & editing:** Lindsay Talemal, Brian Monahan, Juan Poggio.

## Competing Interests

 The authors have no financial disclosures/conflict of interests.

## Funding

 This research received no outside funding support.
